# Neighborhood safety and physical inactivity in adults from Curitiba, Brazil

**DOI:** 10.1186/1479-5868-9-72

**Published:** 2012-06-12

**Authors:** Cassiano Ricardo Rech, Rodrigo Siqueira Reis, Adriano Akira Ferreira Hino, Ciro Romélio Rodriguez-Añez, Rogério Cesar Fermino, Priscila Bezerra Gonçalves, Pedro Curi Hallal

**Affiliations:** 1 Pontifícia Universidade Católica do Paraná, School of Health and Biosciences, Physical Activity and Quality of Life Research Group (GPAQ), Curitiba, Brazil; 2 Federal University of Parana, Curitiba, Brazil; 3 Federal Technological University of Parana, Curitiba, Brazil; 4 State University of Ponta Grossa, Ponta Grossa, Brazil; 5 Federal University of Pelotas, Pelotas, Brazil

**Keywords:** Safety, Physical activity, Adults, Environment

## Abstract

**Background:**

Neighborhood safety is one of the environmental aspects that can influence physical activity. We analyzed the association between perceived neighborhood safety and physical inactivity (PI) in adults and examined effect modification according to sociodemographic variables.

**Methods:**

A cross-sectional study was conducted with 1,261 adults (62% women), age 18–69 years from Curitiba, Brazil.

**Results:**

The perception of unsafe neighborhood was higher among women, older participants, those classified in the high socioeconomic (SES) group, overweighed and also among those reporting to have PA equipments and children. The association between perception safety of walking during the day and walking for leisure (women PR = 1.12 CI_95%_ = 1.02–1.22; men PR = 0.82 CI_95%_ = 0.64–1.05; interaction term PR = 1.38 CI_95%_ = 1.03–1.83) and safe perception was associated with PI, just in the highest SES group (PR = 1.09; CI_95%_ = 1.00–1.19; p trend = 0.032) when compared with their counterparts (low SES PR = 0.99; CI_95%_ = 0.90–1.04; p trend = 0.785; interaction term PR = 1.09; CI_95%_ = 1.03–1.15; p trend = 0.007).

**Conclusion:**

The perception of safety in the neighborhood was associated with PI in transport, but this association varies across of sociodemographic variables.

## Introduction

Physical inactivity (PI) is the 4^th^ leading risk factor for chronic diseases and premature mortality [1]. Inactive individuals have a higher incidence of death from cancer, heart disease and stroke [1]. In Brazil, approximately 15% of adults are physically inactive, and only 31% meet the minimum recommendations for global physical activity [2]. There is consensus that it is necessary to promote strategies to encourage individuals to become more active [3] because regular physical activity (PA), even at moderate levels, such as brisk walking for 30 minutes five or more days a week, can reduce the risk of mortality and morbidity [4]. Reducing physical inactivity can also promote better health in adults [5].

Characteristics of the physical and social environment may contribute to physically inactive behavior [6]. Individuals living in areas with limited access to places for PA, poor lighting, poor quality sidewalks and places with social disorder (e.g., the presence of drugs, crime and robberies) are less active [7]. The association between perceived neighborhood safety and inactivity has been investigated in different countries [8,9], but the results of these studies are inconsistent and have not shown sufficient evidence of an association. Studies conducted in Brazil have also shown inconsistent results [10,11]. Amorim et al. [10] reported a positive association between perceived neighborhood safety and PA during leisure time, but no association with walking for transportation purposes among adults living in southern Brazil.

This inconsistency in findings is due, in part, to different safety indicators used in previous studies and to the fact that most studies have not verified the effect modification of socio-demographic variables, such as gender, age and income. Some investigations have reported that women and elderly and low-income individuals are more likely to perceive low levels of safety in neighborhoods, which can influence their PA [7]. We hypothesize that individuals with lower perceptions of neighborhood safety (e.g., women and the elderly) are more likely to be inactive. For example, the presence of home equipment is positively associated with PA [12]. We hypothesized that people who perceive their neighborhood as less safe invest in equipment for PA practices at home (e.g., treadmills and stationary bikes). Thus, an analysis stratified by gender, income and other demographic or behavioral variables may contribute to the understanding of the relationship between perceived neighborhood safety and physical inactivity.

In Brazil, safety from crime is a serious issue. It is estimated that approximately 77% of adults are afraid of being robbed or murdered, and 75% do not trust the public safety system (e.g., police, police agencies) [13]. An analysis of the impact of environmental characteristics on levels of PA conducted in 11 countries showed that perceptions of crime and a lack of security in walking at night were higher in Brazil (65.5%) and Colombia (74.8%) compared with the United States (31.5%) and Canada (16.1%) [14]. Hence, studies on the association between PI and perceptions of neighborhood safety may contribute to design strategies for interventions in environments with higher social vulnerability.

The primary aim of this study was to determine the association between perceived neighborhood safety and physical inactivity in a sample of adults from Curitiba, Brazil. Our secondary aim was to test the effect modification of gender, income, PA equipment at home and the use of private transport.

## Methods

This primary aim of this cross-sectional study was to verify the association between the utilization of public open spaces and quality of life among Brazilian adults [15]. Curitiba is a state capital in southern Brazil with a population of 1,746,896 inhabitants (52% women) and is the 8^th^ largest city in the country. The city is recognized for its health promotion policies and special attention to green spaces as a means of sustainable development. To date, Curitiba has 19 parks (18,707,232 m^2^), 34 preservation areas (19,378,285 m^2^) and 447 plazas (2,750,740 m^2^) dispersed among 75 neighborhoods [16].

Despite the high quantity of parks and plazas, some places are not intended for physical activity. Locations were selected according to their potential for PA practices and were located in neighborhoods with different economic and environmental conditions so that participants would be representative of the adult population of the city. To select the study locations (parks and plazas), in the first phase, all 75 neighborhoods of the city were classified into nine strata based on a built and social environment (ENV) index for PA and income levels. The built environment information included park density (km^2^/inhabitants), plaza density (km^2^/inhabitants), bike lane density (km^2^/inhabitants), and sports and leisure department units (units/inhabitants). Crime rate (crimes/inhabitants) and traffic accident (deaths/inhabitants) data were used as social environmental indicators. Socioeconomic status (SES) was determined based on median family income [17].

Tertiles for each score (built and social environment and income) were produced and compared in a matrix allowing the neighborhoods to be classified into nine different strata (high, medium and low environments related to PA practice and high, medium and low SES) [17]. Neighborhoods located in the four extreme clusters (high ENV and high income; high ENV and low income; low ENV and high income; and low ENV and low income) were screened to identify eight public open spaces for leisure (4 parks and 4 plazas) [18]. More details on this selection process are available elsewhere [19].

A 500-meter buffer was defined around each of the eight public locations, and all streets within this buffer were audited (n = 1,899). Twenty-nine percent of the street segments were not residential and were excluded from the study (n = 361). One residence was randomly selected in each of the 1,538 eligible segments to establish geographic representation.

Participants were adults (≥ 18 yrs) who had lived in the neighborhood for at least one year. Participants were randomly selected from all eligible residents within each selected household [20]. Three attempts were made on different days and times to contact subjects. Individuals who did not live in the household (e.g., maids and visitors) or those with severe physical impairments that limited PA practice or with cognitive limitations for understanding the questions were excluded.

The interviews were conducted in 95% of the eligible segments (n = 1,461). The refusal rate was 7.9% (n = 121). Eight trained interviewers, all females with high school degrees, conducted the interviews following a 30-hour training. Quality control of the data collection was performed by field supervisors who re-interviewed 74 subjects (12.5% of the sample). The study was approved by the Internal Review Board at the Federal University of Pelotas, and the data collection was conducted between April and July 2009.

The information on perceptions of neighborhood safety in was based on three questions derived from the *Neighborhood Environmental Walkability Scale* ( *NEWS*) [21], translated into Portuguese [22] and adapted for use in Brazil. [10,23,24] The following questions were used: *Are there many crimes in your neighborhood?; Is it safe to walk during the day in your neighborhood?;* and *Is it safe to walk during the night in your neighborhood?* The responses were dichotomized to increase clarity and understanding, and adequate test-retest reliability was obtained (overall agreement ≥84% and kappa ≥0.46; *p* < 0.001). A score was computed by summing the three questions to provide a global measure of neighborhood safety perception. The score ranged from 0 to 3, with “zero” indicating a very safe neighborhood and “3” indicating a very unsafe neighborhood.

The long version of the International Physical Activity Questionnaire (IPAQ) [25], translated and validated for use in Brazil [26], was used to measure PA. Only the leisure and transportation modules were used in this study. Subjects reported their weekly frequency and time spent walking, performing moderate and vigorous intensity PA (MVPA) for leisure and walking for transportation in a typical week [17]. PI was defined as performing “zero” min/wk for each category of PA (walking and MVPA for leisure and walking for transportation).

Age was grouped into five categories (18–29, 30–39, 40–49, 50–59 and ≥ 60 yrs). Body mass index (BMI) was computed based on self-reported information on body mass and height and was categorized into two categories (normal: BMI ≤24.9 km/m^2^ and overweight: BMI ≥ 25 kg/m^2^). Individual SES (“high”, “intermediate” and “low”) was based on the number of assets within the household (e.g., television, washing machine) and educational level. Marital status was grouped into two categories (“single, separated or widower” and “married or living with someone”). Progeny was classified into two categories (having and not having). The use and frequency of private transportation was classified into three categories (“zero d/wk”, “1–5 d/wk” and “6–7 d/wk”). The presence of equipment for PA practice at home [27] was classified into two categories (0 and ≥1).

The analytic sample size included 1,262 subjects. Bivariate associations between demographics, nutritional status and home equipment for PA and safety perception variables were tested through the chi-square test for heterogeneity and linear trend. Poisson regressions were used to verify the unadjusted association between demographics, weight status, home equipment for PA, and safety perception variables with PI. The variables significantly associated with PI in the unadjusted analysis were inserted into a multivariate model. The questions related to neighborhood safety perceptions were inserted separately into the model.

Interaction terms were created to identify the effect modification of gender and SES on safety perception variables (crimes in the neighborhood; safe to walk during the day; safe to walk during the night and score for safety perception) on PA (walking and MVPA on leisure and walking for transport). An interaction between home equipment and safety perception was created to test the effect modification of leisure PA (walking and MVPA) and private transport and safety perception variables on walking for transport. A total of 36 interactions were created. Analyses were conducted using STATA 9.2. All analyses used the sampling design through the “*svy”* commands, considering the 500-meter area around each park/plaza as the primary sampling unit.

## Results

Approximately 62% of the participants were female (Table1). More than half of the participants reported crimes in the neighborhood. Less than two out of ten participants considered it unsafe to walk during the day, whereas almost 80% reported that it was unsafe to walk at night. Almost two-thirds of participants were classified as inactive for leisure time, but only one-third were classified as inactive for walking as a means of transportation.

**Table 1:** Sample characteristics

**Variable**	**Categories**	**n**	**%**
Sex	Men	481	38.1
	Women	781	61.9
Age group (ages)	18–29	280	22.2
	30–39	244	19.3
	40–49	287	22.7
	50–59	293	23.2
	≥ 60	158	12.5
Weight status	Normal	649	51.4
	Overweight	613	48.6
Socioeconomic status	High	153	12.1
	Medium	631	50.0
	Low	478	37.9
Marital status	Single	536	42.5
	Married	726	57.5
Children	No	351	27.8
	Yes	911	72.2
Private transport use	None	335	26.5
	1 to 5 days/week	483	38.3
	6 to 7 days/week	444	35.2
Home facilities for PA	None	736	58.3
	≥ 1	526	41.7
Unsafe perception	Crimes	637	50.5
	Walking during the day	201	15.9
	Walking during the night	979	77.6
Safe perception score	0 (more safe)	212	16.8
	1	439	34.8
	2	455	36.1
	3 (less safe)	156	12.4
Physical Inactivity	Walking for leisure	774	61.3
	MVPA for leisure	871	69.0
	Walking for commuting	376	29.8

Curitiba, Brazil (n = 1,262).

PA: physical activity. MVPA: moderate and vigorous PA.

Table2 shows that perceptions of neighborhood safety were lower among women and elderly participants, those in the high SES group, those who were overweight, those who reported having PA equipment at home, and those with children.

**Table 2:** Association between safe perception in the neighborhood and sociodemographic variables

		**Unsafe perception in the neighborhood**								
**Variables**		**Crimes**			**Walking during the day**			**Walking during the night**		
		**n**	**%**	**p**	**n**	**%**	**p**	**n**	**%**	**p**
Sex										
	Men	243	50.3	0.661	59	12.2	**0.005**	347	71.8	**<0.001**
	Women	383	49.0		142	18.2		633	81.0	
Age group (ages)										
	18–29	118	42.1	0.08	32	11.4	**0.001**	206	73.6	**0.001**
	30–39	137	56.1		26	10.7		175	71.7	
	40–49	139	48.3		50	17.4		228	79.2	
	50–59	147	50.2		66	22.5		239	81.6	
	≥ 60	85	53.5		27	17.0		132	83.0	
Marital status										
	Single	258	48.0	0.366	89	16.6	0.575	405	75.4	0.122
	Married	368	50.6		112	15.4		575	79.1	
Weight status										
	Underweight/Normal	322	49.6	0.948	89	13.7	**0.029**	502	77.3	0.874
	Overweight/Obesity	304	49.4		112	18.2		478	77.7	
Socioeconomic status										
	Low	231	48.3	**0.049**	76	15.9		376	78.7	0.462
	Medium	304	48.2		106	16.8		490	77.7	
	High	90	58.8		19	12.4	0.414	113	73.9	
Children										
	Yes	172	49.0	0.818	49	14.0	0.242	255	72.6	**0.010**
	No	454	49.7		152	16.6		725	79.4	
Private transport use										
	None	158	47.2	0.060	54	16.1	0.164	271	80.9	0.226
	1 to 5 days/week	227	47.0		87	18.0		368	76.2	
	6 to 7 days/week	241	54.0		60	13.5		341	76.5	
Home facilities to PA										
	None	357	48.4	0.361	106	14.4	0.081	588	79.8	**0.023**
	≥ 1	269	51.0		95	18.0		392	74.4	

Curitiba, Brazil (n = 1,262).

PA: physical activity.

Individuals who perceived that it was unsafe to walk at night in the neighborhood were 27% less likely to be inactive in walking for transportation when compared with their counterparts. There were no other associations between PI and perceived neighborhood safety after adjusting for potential confounders (Table3).

**Table 3:** Adjusted association between physical inactivity and safety in the neighborhood in adults

**Variable**	**Category**	**Physical inactivity**								
		**Walking for leisure**^**a**^			**MVPA**^**b**^			**Walking for commuting**^**c**^		
		**%**	**PR (CI95%)**	**p**	**%**	**PR (CI95%)**	**p**	**%**	**PR (CI95%)**	**p**
Safety from crime										
	Safe	60.5	1.00		71.3	1.00		30.3	1.00	
	Unsafe	62.2	0.99 (0.94–1.06)	0.858	66.7	1.05 (0.98–1.13)	0.104	29.3	1.11 (0.77–1.60)	0.506
Is safe walk during the day										
	Safe	61.2	1.00		68.5	1.00		30.2	1.00	
	Unsafe	62.2	1.03 (0.95–1.11)	0.363	71.6	0.99 (0.90–1.09)	0.821	27.9	0.96 (0.69–1.34)	0.387
Is safe walk during at night										
	Safe	62.3	1.00		62.9	1.00		38.2	1.00	
	Unsafe	61.1	0.97 (0.82–1.15)	0.741	70.8	1.04 (0.90–1.22)	0.463	27.4	0.73 (0.57–0.94)	**0.023**
Scale of neighborhood safety										
	0 (more safe)	69.2	1.00	0.905*	73.8	1.00	0.271*	58.5	1.00	0.491*
	1	60.4	1.05 (0.84–1.30)	0.610	66.7	1.14 (0.93–1.40)	0.171	28.5	0.98 (0.61–1.57)	0.929
	2	61.9	0.97 (0.81–1.17)	0.734	71.1	1.12 (0.91–1.36)	0.228	28.1	0.87 (0.52–1.44)	0.525
	3 (less safe)	56.4	1.06 (0.94–1.19)	0.275	69.2	1.11 (0.89–1.37)	0.271	25.6	0.94 (0.58–1.52)	0.766

Curitiba, Brazil (n = 1,262).

PA: physical activity.

MVPA: moderate and vigorous PA.

^a^Adjusted for sex, age, SES, private transport use and home facilities to PA.

^b^Adjusted for sex, age, nutritional status, SES, marital status, children, private transport use and home facilities to PA.

^c^Adjusted for sex, age, SES, marital status, private transport use.

*p trend.

There was an effect modification of gender on the association between safety perceptions of walking during the day and walking for leisure (women PR = 1.12, CI_95%_ = 1.02–1.22; men PR = 0.82, CI_95%_ = 0.64–1.05; interaction = 1.38, CI_95%_ = 1.03–1.83) after adjusting for potential confounders.

Lower perceptions of safety were associated with inactivity in the highest SES group (PR = 1.09; CI_95%_ = 1.00–1.19; p for trend = 0.032) when compared with their lowest counterparts (low SES PR = 0.99; CI_95%_ = 0.90–1.04; p for trend = 0.785; interaction term PR = 1.09; CI_95%_ = 1.03–1.15; p for trend = 0.007).

The relationship between perceptions of safety and PI for MVPA showed an effect modification by home equipment for PA. There was a trend toward increased PI for moderate-vigorous activities higher perceptions of a lack of safety (PR = 1.05; CI_95%_ = 1.00–1.11; p trend = 0.04), but only for participants who reported not having facilities for PA at home (interaction term PR = 0.93 CI_95%_ = 0.85–1.01; *p* = 0.07).

We observed that a higher perception of a lack of safety for walking at night was associated with a lower risk of inactivity in walking for transportation for all categories of private transport use (6–7 days/wk PR = 0.75, IC_95%_ = 0.60–0.94; 5–6 days/wk PR = 0.84 IC_95%_ = 0.62–1.12; none PR = 0.46 CI_95%_ = 0.29–0.71). However, this relationship differed significantly between those reporting 6–7 days/week (interaction term PR = 1.62, CI_95%_ = 1.11–2.36, *p* = 0.021) and 1–5 days/wk (interaction term PR = 1.91; CI_95%_ = 1.50–2.44; *p* = 0.001) compared with those who did not use private transport.

## Discussion

The aim of this study was to examine the relationship between perceived neighborhood safety and PI according to specific domains and types of activities (walking and performing MVPA for leisure and walking for transportation). The results indicated that associations were domain-specific and varied according to the safety indicator. We also observed that this association was modified by gender, age and SES. It is believed that certain groups are more vulnerable to perceptions of a lack of safety in the neighborhood, which may undermine the relationship with PA [7]. The results of this study support evidence that women and elderly and high SES individuals have lower perceptions of neighborhood safety [7,28].

The relationship between PI and perceptions of safety was found to be complex in this study. After adjusting for confounders, only individuals who perceived that it was unsafe to walk at night in the neighborhood were less likely to be inactive in transport. Similar results were found in studies conducted in Brazil [11,23,29]. The inverse relationship found in this study may be explained by two main reasons. First, individuals who walk more may see more crimes while commuting and therefore may have lower perceptions of safety when compared with individuals who spend more time at home or who walk less. Second, walking for transportation is a utilitarian activity, and some people need to walk even though they perceive the neighborhood as unsafe. This situation results in higher exposure to an unsafe environment. In fact, we found that approximately 80% of individuals perceived their surroundings as unsafe when walking at night. This relationship was modified by the use of private transport (cars). Individuals who regularly used a car (6 to 7 days/week) and considered their neighborhood unsafe were less likely to be inactive.

Multivariate analysis showed no association between the perception of neighborhood safety and PI in leisure time. However, when analyzing the association according to gender, we found that women with higher perceptions of a lack of safety were more likely to be inactive in walking during leisure time than men. This effect modification of gender was observed in other studies that demonstrated a consistent association among women [7,30]. In part, these results may be explained by other psychosocial mediators. For instance, greater perceived self-efficacy among men may increase perceptions of neighborhood safety because evidence suggests an interaction between these factors [31]. Because physical inactivity is usually higher among women, increasing perceptions of neighborhood safety in could be a useful strategy to prevent PI in this group. Evidence indicates that improvements in lighting and aesthetics can contribute to decreasing feelings of a lack of safety and perceptions of crime in neighborhoods [32]. This is an important fact for public health promotion, particularly in Brazil and other areas with a high prevalence of crime. For instance, in Brazil, women lose an average of 43.3 years of life due to homicides [33], which contributes to other social and economic problems in society.

The modified effect of SES on the association between perceived safety and PI has been investigated in different populations [34,35]. We found that individuals with high SES and increased perceptions of insecurity were more likely to be inactive than those with medium or low SES. In general, low-SES individuals are more vulnerable to unsafe neighborhoods [35], but this does not necessarily indicate greater perceptions of a lack of safety because individuals incorporates this insecurity into their daily lives. Thus, it can be inferred that high-SES individuals may report more insecurity, a feeling that may be shared by other members of the group. In this context, social norms may play an important role in neighborhood perceptions of safety.

Finally, these results demonstrate that the relationship between perceived neighborhood safety and PI is complex, and the effect modification of demographic characteristics (e.g., gender, SES) may explain this association. Interventions to increase perceived neighborhood safety are priorities in specific population groups (women, elderly individuals and individuals with high SES). Insecurity is a major barrier to active behavior, yet little is known about the mechanisms of this relationship [7]. We suggest that future studies should examine the indirect effect of perceived neighborhood safety on PI through intrapersonal (self-efficacy, enjoyment) and interpersonal (social support) variables [6].

Some limitations and strengths should be considered for better interpretation and extrapolation of these results. Although the sample is not representative of Curitiba, the sample size is sufficient to detect associations [33]. The sampling design was considered, but this characteristic was not controlled in the analyses. The cross-sectional design does not allow us to draw causal relationships. The measurements of safety and PI were obtained by self-reported measures, so errors in judgment or misinterpretation are expected. Despite these limitations, these measures are commonly used in PA studies and have shown good reliability and reasonable validity. Finally, a strength of this study is the overall process, which included household surveys.

## Conclusion

Physical inactivity in the form of walking for transportation was lower among individuals with high perceptions of unsafe neighborhoods, and this association was stronger among individuals who regularly used private transportation. Other results suggest that women and individuals with high SES are less active in walking for leisure when they perceive a neighborhood as unsafe. It is suggested that further studies should test the effect modification of socio-demographic variables in this relationship and should analyze the indirect effects on perceptions of neighborhood safety of interpersonal and intrapersonal aspects related to PI.

## Competing interests

The authors declare that they have no competing of interests.

## Authors’ contributions

RCR, FRC, GPB, HAAF, and conceived designed the study, prepared the data, conducted the analysis and interpretation of data. RACR, RRS, contributed to study design, data interpretation, and writing. HPC, contributed to study design and revised the manuscript for important intellectual content. All authors read and approved the final.
